# Supplementary material for: Phenotypic Variation and Peel Contribution to Fruit Antioxidant Contents in European and Japanese Plums
Source: Plants (Basel). 2022 May 18;11(10):1338. doi: 10.3390/plants11101338 (PMC9143520; doi:10.3390/plants11101338)
Supplement: Supplementary file 1 [file plants-11-01338-s001.zip › Figure S1 and S2.pdf]

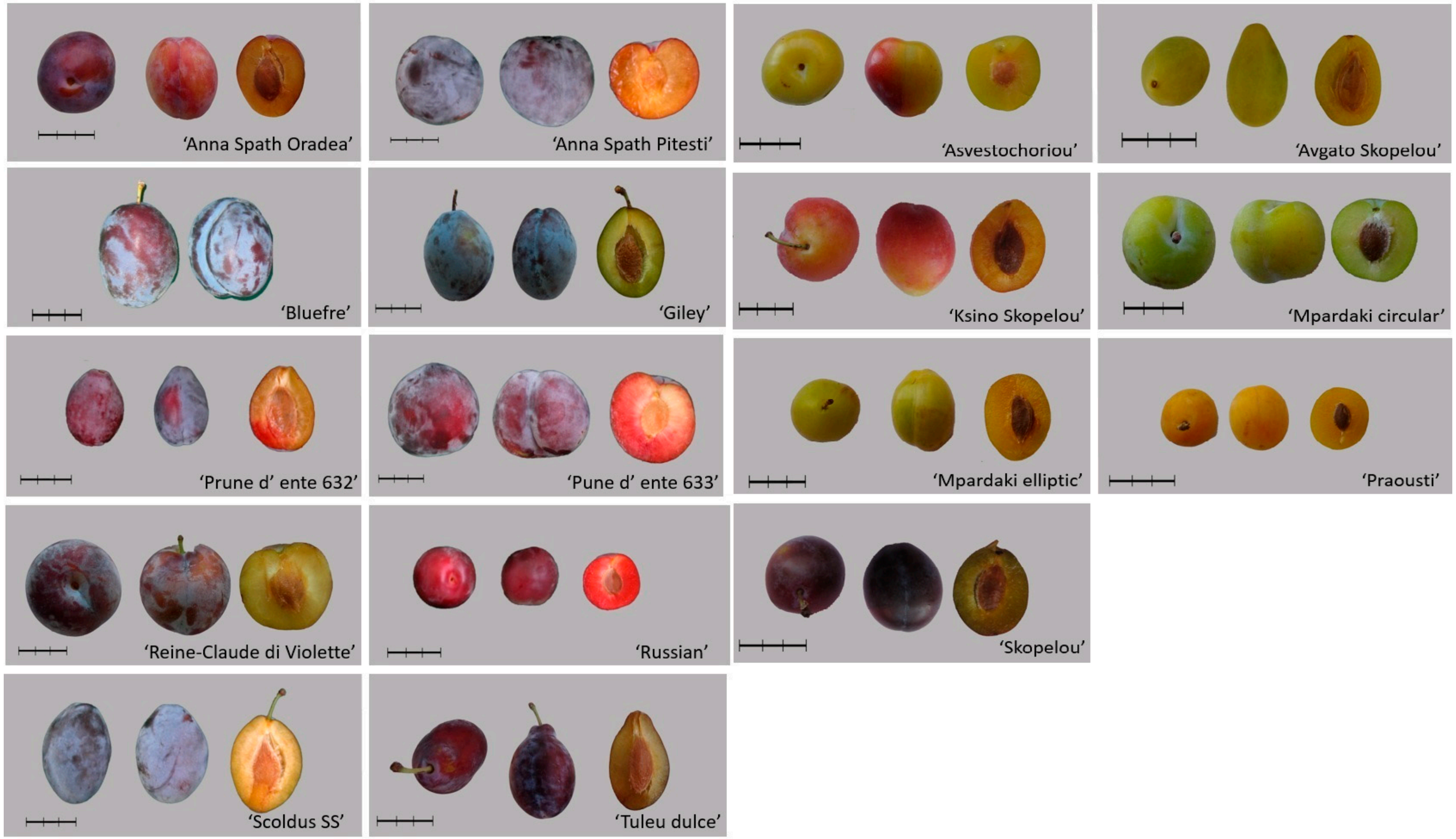

Figure S1. Fruit from local and foreign European plum cultivars studied.

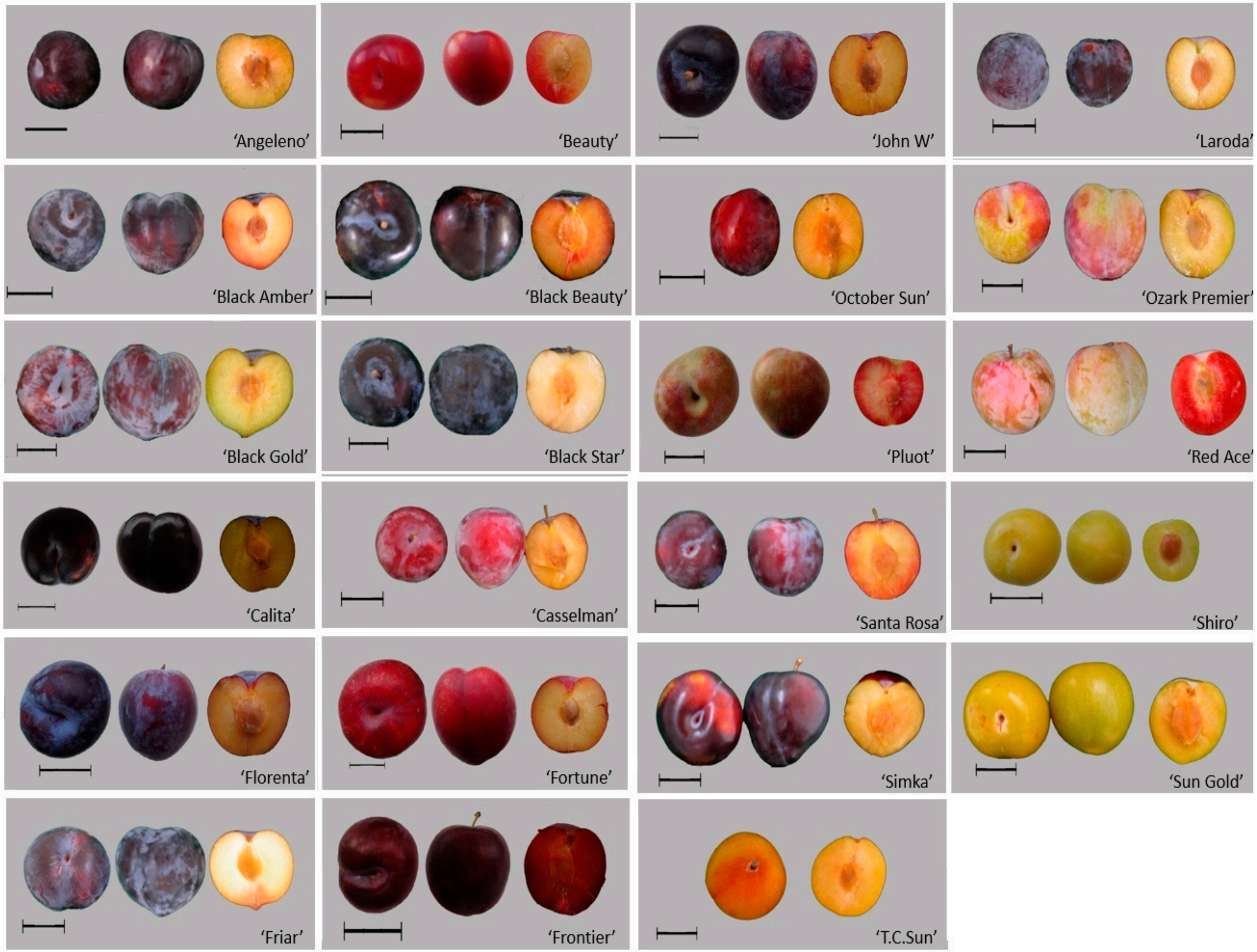

Figure S2. Fruit from the Japanese plum cultivars studied.
