# Supplementary material for: Phenotypic Variation and Peel Contribution to Fruit Antioxidant Contents in European and Japanese Plums
Source: Plants (Basel). 2022 May 18;11(10):1338. doi: 10.3390/plants11101338 (PMC9143520; doi:10.3390/plants11101338)
Supplement: Supplementary file 1 [file plants-11-01338-s001.zip › Manuscript plum TABLES Suppl.pdf]

Table S1. Color CIELAB parameters measured in fruit skin and flesh tissue of 19 European and 24 japanese plum cvs grown under the same experimental conditions.

	L*-peel	a*-peel	b*-peel	h*-peel	Chroma-peel	L*-flesh	a*-flesh	b*-flesh	h*-flesh	Chroma-flesh
European cvs										
AnnaSpath Oradea	38.6	14.9	-1.2	177.0	15.6	55.4	-0.9	20.3	92.6	20.4
AnnaSpath Pitetsi	32.0	9.5	-10.5	310.5	14.7	37.4	4.6	11.6	66.4	12.7
Asvestochoriou	48.8	14.6	16.5	49.7	24.9	57.7	6.7	28.0	76.6	28.9
Avgata Skopelou	51.8	5.0	32.4	81.4	32.8	47.2	1.1	30.5	87.8	30.6
Bluefre	22.5	4.7	4.7	44.5	6.7	56.0	5.4	36.9	81.8	37.3
Giley	32.0	6.5	6.4	44.7	9.1	49.9	0.9	31.7	88.3	31.8
Ksina Skopelou	47.9	20.4	20.1	44.8	28.9	52.1	2.7	41.2	86.2	41.3
Mpardaki Circular	46.5	-2.2	41.9	93.0	42.0	53.7	2.3	34.7	86.3	34.8
Mpardaki Elliptic	50.7	-0.1	44.9	90.1	45.0	54.5	3.4	36.0	84.7	36.2
Praousti	57.4	1.1	44.4	88.6	44.5	57.2	1.6	37.1	87.5	37.1
President	26.2	11.7	9.6	38.8	15.2	50.8	3.4	34.6	84.4	34.8
Prune d'ente 632	36.2	17.3	0.4	273.6	19.1	44.6	2.9	17.0	79.0	17.6
Prune d'ente 633	36.8	15.8	-2.3	259.0	17.5	49.6	18.9	7.7	25.6	21.0
Reine-Claude di Violette	38.5	18.2	3.5	101.7	18.8	51.6	0.6	16.0	89.3	16.4
Russian	26.4	21.5	-4.0	319.0	21.9	37.5	34.2	12.0	19.2	36.3
Scoldus SS	31.4	7.0	-9.9	305.2	12.5	59.0	1.6	19.6	85.6	19.6
Skopelou	34.5	6.2	0.5	176.1	7.0	40.1	-3.1	25.7	97.5	26.0
Stanley	32.1	4.4	-11.7	290.7	12.6	55.1	0.5	19.7	88.3	19.7
Tuleu Dulce	35.0	10.6	-9.1	290.5	15.5	50.3	4.2	15.7	72.8	16.6
Japanese cvs										
Angeleno	24.0	6.7	6.6	45.5	9.4	58.4	1.5	29.3	87.1	29.4
Autumn Giant	38.4	27.6	2.7	125.0	28.3	61.2	5.7	22.1	76.0	22.9
Beauty	33.0	25.1	17.1	34.0	31.0	49.4	4.6	30.0	81.5	15.2

Black Amber	25.7	5.4	-9.3	299.8	11.0	60.8	5.2	16.1	71.6	17.0
Black Beauty	21.3	10.6	9.9	44.1	14.6	38.6	18.3	23.1	52.1	29.7
Black Gold	29.9	14.4	-6.0	337.0	15.9	56.6	-0.6	17.1	92.3	17.2
Black Star	28.0	7.1	-7.6	310.0	10.9	61.6	0.7	8.3	84.9	8.4
Calita	29.2	18.7	-3.4	287.5	19.5	55.9	-0.3	16.3	91.1	16.4
Casselman	32.3	20.1	-3.2	288.8	20.7	48.6	3.7	17.4	77.5	18.1
Florentia	23.0	18.6	11.3	31.2	21.8	48.7	4.7	34.4	82.3	34.8
Fortune	43.1	28.3	9.6	20.3	30.8	61.7	0.1	22.5	89.8	22.5
Friar	27.7	8.3	-7.7	314.8	11.8	64.3	-1.4	15.7	95.3	15.8
Frontier	32.4	14.3	-5.8	335.9	15.8	46.6	23.8	8.6	20.5	25.4
John W	34.4	16.7	-10.0	327.9	20.4	58.8	4.1	19.1	78.0	19.6
Laroda	33.5	18.2	-2.1	259.3	19.4	66.1	-0.2	20.7	90.9	20.8
October Sun	53.1	15.3	18.6	52.7	28.5	49.7	6.6	23.5	74.3	24.5
Ozark Premier	40.1	23.7	5.8	131.0	25.8	60.5	2.2	20.2	84.2	20.4
Pluot	29.9	10.0	-8.0	319.8	13.1	55.0	8.0	14.5	61.8	17.5
Red Ace	55.3	3.7	21.3	78.9	23.2	53.7	19.4	24.8	51.9	32.0
Santa Rosa	29.5	14.0	-6.5	332.8	15.8	48.6	5.1	13.7	70.7	15.3
Shiro	50.4	0.7	36.2	88.4	36.2	53.3	0.5	44.3	89.2	44.3
Simka	33.2	18.6	-2.1	232.0	19.2	62.4	1.1	18.5	86.5	18.6
Sun Gold	59.7	-2.9	31.4	95.2	31.7	48.2	3.5	18.1	78.8	18.4
T.C. Sun	58.4	1.6	28.2	86.7	28.6	58.8	3.0	20.1	82.0	20.4

Supplementary Table S2. Leaf shape (1, ovate; 2, elliptic; 3, obovate), leaf tip shape (1, acute; 2, right-angled; 3, obtuse), shape of base (1, acute; 2, obtuse; 3, truncate) and dimensions of leaf blade length (LBL), leaf blade width (LBW), stalk length (SL) (cm) and ratio of LBL/LBW and LBL/SL in European and Japanese plum cvs.

	Leaf shape	Leaf tip shape	Shape of base	LBL	LBW	SL	LBL/LBW	LBL/ SL
European cvs								
AnnaSpath Oradea	3	2	2	7.9	4.7	1.3	1.7	6.2
AnnaSpath Pitetsi	2	1	1	9.3	4.4	1.1	2.1	8.7
Asvestochoriou	2	1	1	12.0	5.4	1.2	2.2	10.0
Avgata Skopelou	2	1	1	9.7	6.0	2.2	1.6	4.5
Bluefre	3	3	2	8.3	5.8	1.5	1.4	5.8
Giley	1	1	2	9.4	5.1	2.0	1.9	5.2
Ksina Skopelou	3	3	2	6.9	4.6	1.8	1.5	3.8
Mpardaki Circular	3	3	1	7.3	4.2	1.4	1.7	5.2
Mpardaki Elliptic	3	3	1	9.1	6.1	1.6	1.5	5.7
Praousti	2	2	1	5.6	3.5	1.3	1.6	4.5
President	1	2	2	9.2	5.7	1.5	1.7	6.5
Prune d'ente 632	3	2	2	8.2	5.2	1.9	1.6	4.6
Reine-Claude di Violette	1	3	3	10.0	6.9	1.7	1.5	6.2
Scoldus SS	2	1	2	7.8	4.1	2.1	1.9	3.8
Skopelou	2	1	1	10.3	6.9	2.6	1.5	4.0
Stanley	1	2	2	7.9	5.5	1.9	1.4	4.2
Tuleu Dulce	1	2	2	10.2	6.3	1.9	1.6	5.6
Japanese cvs								
Angeleno	2	1	1	9.2	4.8	1.3	1.9	7.2
Autumn Giant	2	1	1	9.9	4.3	1.5	2.5	7.1
Beauty	2	1	1	9.0	3.5	1.2	2.6	7.4
Black Amber	2	1	1	9.3	3.0	1.3	8.7	7.4
Black Beauty	2	1	1	8.5	4.0	1.1	2.2	7.7
Black Gold	2	1	1	8.8	4.0	1.3	2.2	6.9
Black Star	2	1	1	12.2	4.9	1.2	2.6	10.7
Calita	2	1	1	11.0	4.1	2.2	2.7	5.1
Casselman	2	1	1	12.8	5.6	1.9	2.3	7.0
Florentia	2	1	1	9.3	3.5	1.3	2.7	7.2
Fortune	2	1	1	11.0	5.4	1.1	2.0	9.8
Friar	2	1	1	8.6	3.5	1.3	2.5	6.5
Frontier	2	1	1	10.0	3.1	1.8	3.2	5.5
John W	2	1	1	9.0	3.6	1.0	2.6	9.1
Laroda	2	1	1	8.2	3.6	1.3	2.3	6.7
October Sun	2	1	1	9.3	3.7	1.5	2.6	6.5
Ozark Premier	3	3	1	9.6	4.3	1.4	2.2	7.1
Pluot	1	2	2	9.3	5.2	1.3	1.8	7.0
Red Ace	2	2	1	9.5	4.6	1.4	2.1	7.2
Santa Rosa	3	3	1	10.3	4.5	1.4	2.3	7.4
Simka	2	1	1	8.0	4.0	1.1	2.0	7.3
Sun Gold	2	1	1	10.1	4.2	2.2	2.4	4.8

T.C. Sun	2	1	1	10.9	4.7	1.3	2.3	8.7
